# An analysis of fatal adverse conditions in temporal association of COVID-19 vaccination to boost the safety of vaccination for COVID-19

**DOI:** 10.1186/s43162-023-00191-7

**Published:** 2023-02-07

**Authors:** Shahnawaz Muslim, Gulam Mustafa, Nasrin Nasrin, Aaisha Firdaus, Shambhu Kumar Singh

**Affiliations:** 1 Madhubani Medical College and Hospital, Madhubani, Bihar India; 2 College of Pharmacy, Shaqra University, Shaqra, 11961 Saudi Arabia; 3 Katihar Medical College & Hospital, Katihar, Bihar India; 4 Eiwa Hospital, Katihar, Bihar India

**Keywords:** COVID-19 vaccines, Vaccination and death, COVID-19, SARS-CoV-2

## Abstract

SARS-CoV-2, the causative agent of COVID-19, claimed multiple lives in a very short span of time. Seeing the urgency of situation, vaccines were developed in hitherto unseen time frame. Vaccines definitely passed the test of safety and efficacy in clinical trials, but post mass vaccination data revealed cases of fatal adverse conditions in the temporal association of vaccination.

The temporal association does not guarantee that the fatality is due to vaccination, but at the same time, it does create a concern. To overcome this concern and improve the safety of vaccination, we reviewed literature and collected data of 15 studies comprising of total 22 cases of fatal adverse condition/death in the temporal association of COVID-19 vaccination.

Analysis of these data shows that many persons (40.90%) who succumbed were previously healthy individuals. All those who died developed symptoms or were admitted to hospital within a period of 3 weeks after vaccination. 86.36% cases of death took place within a period of 3 weeks after vaccination/presentation/admission/intervention. Complications which lead to death were CVST, thrombocytopenia/thrombosis /VITT, DIC and haemorrhage in 81.18% of cases. 81.81% cases of death were noted in the temporal association with ChAdOx1 nCoV-19 vaccine. 68.18% persons developed symptoms after first dose. Death was more common in females (59.09%), and the most commonly affected age group was 20 to 60 years (86.36%).

Knowledge of fatal adverse conditions in the temporal association of vaccination will help to tackle these situations well and improve the safety of vaccination drive further.

## Background

COVID-19, a disease caused by SARS-CoV-2, has drastically changed the life of human being in last 2 years. All around the world, fear of surge of COVID-19 cases, development of new variant, getting infected with virus and loss of own/near and dear one’s life keeps looming. In order to combat this viral disease, old time-tested formula of vaccine was brought forward. But, the time to test the vaccine, i.e. time required from identification of virus to development of vaccine to approval of vaccine, was kept short.

While the time required from isolation/identification of pathogen in laboratory to approval of vaccine for mass vaccination programme for most of the vaccines is in years, this time frame for COVID-19 vaccines is within a year. Fastest time in which a vaccine got approval after sampling of virus is 4 years, and the vaccine which got the approval in this time range is mumps vaccine developed in 1960s [1].

Though COVID-19 vaccines have been found to be safe and effective in clinical trials, a close watch on adverse effects developing after vaccination is essential as large numbers of people are being vaccinated and even a small fraction of serious adverse effect may be a significant number.

Most of the adverse effects developing after vaccination have been found to be mild and self-limiting. However, some cases of serious adverse effects after vaccination have also been reported. Few reports of adverse effects which ultimately resulted into death have also been found. Though cause of death in these reports cannot be directly attributed to vaccination, the temporal association with vaccination does raise the question [2–16].

In this study, an attempt has been made to present information about fatal adverse effects developing after vaccination (whether causal or merely an association), so that a better understanding of these lethal untoward effects can be made and appropriate measures to handle these adverse situations can be taken and ultimately safety of vaccination can be improved.

## Material and methods

In order to find out cases of death after COVID-19 vaccination, literature search was performed using the key words like death/mortality/adverse effects after COVID-19 vaccination. Since many reports of serious adverse effects like myocarditis and thrombotic thrombocytopenia after COVID-19 vaccination were noted, these papers were also thoroughly evaluated to find cases who succumbed to death. Only case reports and case series were included in the study. A total of 15 studies with 22 deaths in the temporal association of COVID-19 vaccination were noted.

## Result

Data was collected from 15 different studies for 22 cases of death in the temporal association of COVID-19 vaccination (see Table 1); when analysed in relation to various factors like adverse effects after vaccination which probably lead to death, past medical history, age and sex of person vaccinated, type of vaccine used, time of onset of symptoms/admission after vaccination, dose after which symptoms developed, and time of death after presentation/vaccination/admission/intervention, it yielded the following results.

**Table 1:** Showing features of death in the temporal association of COVID-19 vaccination

S. no	Study	Adverse effect	Age	Sex	Previous med history	Type of vaccine	Time of onset of symptoms/admission after vaccination	Dose after which symptoms developed or Pnt needed admission	Outcome
1	Tajastra et al. [2]	Acute coronary thrombosis/myocardial infarction	86 years	M	Previous H/O Ca prostate Status—post surgery and RT On anti-androgen therapy H/O atrial fibrillation	Pfizer BioNTech	After 30 min of injection	1st dose	Death (3 days after vaccination)
2	Blaunfeldt et al. [3]	Thrombocytopenia with acute ischemic stroke and bleeding (B/L adrenal haemorrhage)	60 years	F	H/o Hashimoto’s thyroiditis +HTN	AZD1222	After 7 days	1st dose	Death (on 6th day of admission)
3	D’Agostino [4]	Cerebral venous thrombosis and disseminated intravascular coagulation	54 years	F	H/o Ménière’s disease	AstraZeneca	12 days	1st dose	Death (after 5 days of admission)
4	Fahmida Bano [5]	Intracerebral haemorrhage and C VST	53 years	F	H/o fibromyalgia	ChAdOx1 nCoV-19	14 days	1st dose	Death (on 16th day after vaccination)
5	Fahmida Bano [5]	CVST and subarachnoid haemorrhage	55 years	M	NAD	ChAdOx1 nCoV-19	10 days	1^st^ dose	Death (within 24 h of presentation)
6	Rela [6]	Autoimmune hepatitis	62 years	M	H/o DM, past H/o jaundice	Covishield	16 days	NA	Death (3 weeks after admission)
7	Mahmood Nasar [7]	Myocarditis	70 years	F	H/o multiple sclerosis	Janssen COVID-19 vaccine	2 days	NA	Death (on 8th day of admission)
8	Preethi suresh [8]	VITT and CVST	27 years	M	NAD	ChAdOx1 nCoV-19	48 h	1st dose	Death (time from admission to death = NA)
9	Castelli et al. [9]	CVST and thrombocytopenia	50 years	M	NAD	AstraZeneca COVID vaccine	7 days	1st dose	Death (48 h after admission)
10	Tor Halvor [10]	Intracerebral haemorrhage	Thirties	F	NAD	ChAdOx1 nCoV-19	7 days	NA	Death (died the following day of admission)
11	Massimo Franchinia [11]	CVST	50 years	M	NAD	AstraZeneca vaccine	7 days	1st dose	Death (18 h after intervention)
12	Mehta P [12]	CVST and thrombocytopenia	32 years	M	NAD	Vaxzevria vaccine	9 days	1st dose	Death (time from admission to death = NA)
13	Mehta p [12]	CVST and thrombocytopenia	25 years	M	H/o primary sclerosing cholangitis and migraine	Vaxzevria vaccine	6 days	1st dose	Death (time from admission to death = NA)
14	Gessler [13]	CVT (cerebral sinus and vein thrombosis)	47 years	F	NAD	ChAdOx1 nCoV-19	12 days	1st dose	Death (39 h after admission)
15	Gessler [13]	CVT (cerebral sinus and vein thrombosis)	50 years	F	NAD	ChAdOx1 nCoV-19	7 days	1st dose	Death (49 h after admission)
16	Gessler [13]	CVT (cerebral sinus and vein thrombosis)	44 years	F	.NAD	Ad26.COV2.S	10 days	1st dose	Death (20 h after admission)
17	Wiedmann M [14]	VITT and CVT (cerebral venous thrombosis)	34 years	F	H/o pollen allergy H/o contraceptive vaginal ring	ChAdOx1 nCoV-19	7 days	NA	Death (day after admission)
18	Wiedmann M [14]	VITT and CVT (cerebral venous thrombosis)	42 years	F	H/o pollen allergy H/o contraceptive vaginal ring	ChAdOx1 nCoV-19	10days	NA	Death (on day 15 after admission)
19	Wiedmann M [14]	VITT and CVT (cerebral venous thrombosis)	37 years	F	H/o pollen allergy H/o oral contraceptive	ChAdOx1 nCoV-19	8 days	NA	Death (on day 11 after vaccination)
20	Wiedmann M [14]	VITT and CVT (cerebral venous thrombosis)	54 years	F	H/o HTN. H/o HRT	ChAdOx1 nCoV-19	6 days	NA	Death (2 days after surgery/craniotomy)
21	Aladdin Y [15]	VITT and DIC	36 years	F	H/o DM	ChAdOx1 nCoV-19	14 days	1st dose	Death (4 days after admission)
22	Choi S [16]	Myocarditis	22 years	M	H/o HTN	BNT162b2 mRNA	5 days	1st dose	Death (few hours after admission)

*CVST* Cerebral venous sinus thrombosis, *VITT* Vaccine-induced thrombotic thrombocytopenia, *DIC* Disseminated intravascular coagulation, *HTN* Hypertension, *NAD* Nothing abnormal detected, *DM* Diabetes mellitus, *NA* Not available

### Adverse effects noted after vaccination

CVST was the most common complication resulting into death. Out of 22 deaths, it was found to be associated with 15 (68.18%) cases of death. In majority of cases, CVST was associated with thrombocytopenia (*n* = 8). It was also found to be associated with DIC, intracerebral haemorrhage, and SAH.

Thrombocytopenia with acute ischemic stroke with B/L adrenal haemorrhage, intracerebral haemorrhage, VITT with DIC, and autoimmune hepatitis; each resulted in one case of death (4x 4.54%). Myocardial infarction was noted in one case (4.54%) and myocarditis in 2 cases (9.09%) of death (see Table 2).

**Table 2:** Adverse effects noted after COVID-19 vaccination

S.no.	Adverse effect	No. of cases
1.	CVST with DIC	1
2.	CVST with intracerebral haemorrhage	1
3.	CVST with SAH	1
4.	CVST with thrombocytopenia	8
5.	CVST	4
6.	Thrombocytopenia with acute ischemic stroke and adrenal haemorrhage	1
7.	Intracerebral haemorrhage	1
8.	VITT and DIC	1
9.	Myocarditis	2
10.	MI	1
11.	Autoimmune hepatitis	1
Total 22		

*CVST* Cerebral venous sinus thrombosis, *DIC* Disseminated intravascular coagulation, *SAH* Subarachnoid haemorrhage, *VITT* Vaccine-induced thrombotic thrombocytopenia, *MI* Myocardial infarction

### Past medical history

Out of 22 cases, majority of cases (*n* = 9) (40.90%) had no significant previous medical history. Three (13.63%) patients had H/o HTN out of which one had H/o HTN along with Hashimoto thyroiditis, one had HTN and H/o HRT, and one patient had only HTN. Diabetes mellitus was noted in 2 cases (9.09%), out of which one had DM and past H/o jaundice and one had only DM. H/o pollen allergy was found in 3 cases (13.63%) out of which 2 patients also had H/o contraceptive vaginal rings and one patient had H/o OCP along with pollen allergy. Past H/o Ca prostate (status—post sx and RT) on ADT along with h/o atrial fibrillation was found in one case (4.54%). Individuals with H/o Ménière’s disease (1 case = 4.54%), fibromyalgia (1 case = 4.54%), multiple sclerosis (1 case = 4.54%), and H/o PSC along with migraine (1 case = 4.54%) accounted for the remaining cases (see Table 3).

**Table 3:** Past medical history of persons who succumbed to death

S.no	Past medical history	No. of cases
1.	NAD	9
2.	HTN	3 (HTN with HT = 1 HTN with H/o HRT = 1 HTN alone = 1)
3.	DM	2 (DM + past H/o jaundice = 1 DM = 1)
4.	H/o pollen allergy	3 (H/o pollen allergy + VCR = 2 H/o pollen allergy + OCP = 1)
5.	H/o Ca prostate (status—post Sx + RT on ADT + H/o AF)	1
6.	H/o Ménière’s disease	1
7.	H/o fibromyalgia	1
8.	H/o multiple sclerosis	1
9.	H/o PSC+ migraine	1
Total= 22		

*NAD* Nothing abnormal detected, *HTN* Hypertension, *DM* Diabetes mellitus, *HT* Hashimoto thyroiditis, *HRT* Hormone replacement therapy, *VCR* Vaginal contraceptive ring, *OCP* Oral contraceptive pill, *Sx* Surgery, *RT* Radiation therapy, *ADT* Androgen deprivation therapy, *AF* Atrial fibrillation

### Age and sex wise distribution

Most of the deaths (19 out of 22 = 86.36%) took place between ages 20 to 60 years.

**Fig. 1:**
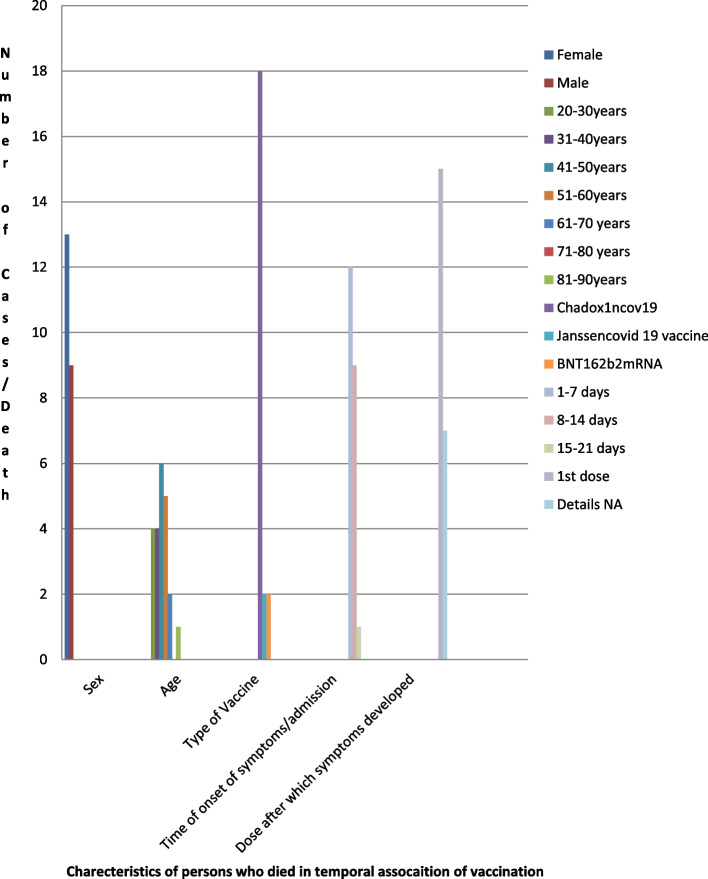
Characteristics of persons who died in the temporal association of vaccination along with number of deaths

Females outnumbered the males. Out of 22 cases, 13 were females (59.09%), and 9 were males (40.90%) (see Fig. 1).

### Type of vaccine

Eighteen out of 22 cases (= 81.81%) were associated with ChAdOx1 nCoV-19 vaccine {this includes ChAdOx1 nCoV-19 vaccines (11) +AstraZeneca vaccine (3) + Covishield (1) + Vaxzevria (2) + AZD-122(1)}. BNT 162 b2 MRNA was associated with 2 deaths (9.09%). Janssen COVID-19 vaccine was associated with two cases (9.09%) (see Fig. 1).

### Time of onset of symptoms/admission

Most of the cases, 12 out of 22 (54.54%), developed symptoms or were admitted to hospital within 7 days of vaccination. Nine cases (40.90%) were noted in 2nd week and one case (4.54%) in 3rd week. Earliest onset of symptom was within 30 min (noted in one case) (see Fig. 1).

### Dose after which symptoms developed

Fifteen cases (68.18%) developed symptoms after 1st dose. In 7 cases (31.81%), exact description of dose could not be made out (see Fig. 1).

### Time of death after vaccination/presentation/admission/intervention

**Fig. 2:**
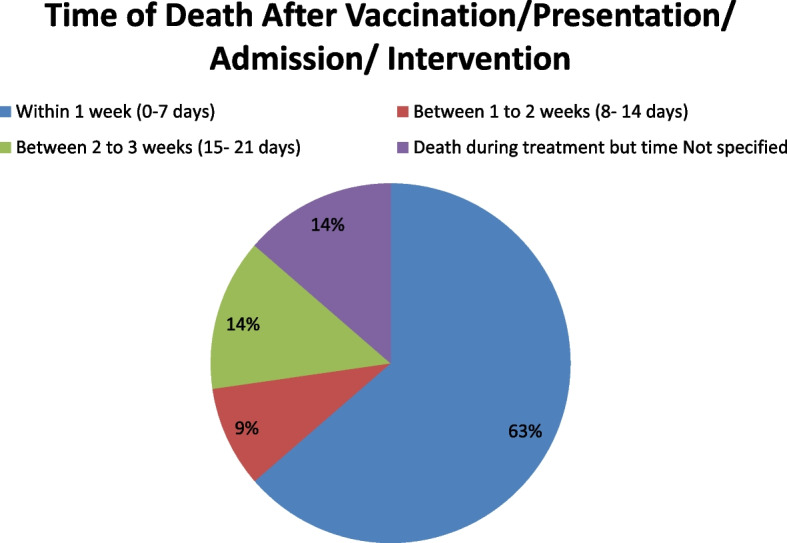
Time of death after vaccination/ presentation/admission/intervention along with number of deaths during that time frame

Majority of death (14 out of 22 = 63.63%) took place within 1 week after vaccination/presentation/admission/intervention. Out of these 14 cases, 11 deaths occurred within 3 days. Two deaths (9.09%) occurred between 1 to 2 weeks and 3 deaths (13.63%) between 2 to 3 weeks. In 3 cases (13.63%), the exact time of death after presentation/vaccination/admission/intervention could not be identified, but these deaths took place during treatment for the complication developed after vaccination (see Fig. 2).

## Discussion

SARS-CoV-2, the causative agent of COVID-19 starting from Wuhan China, spread all around the globe in very short period of time. The impact of the disease all around the world is palpable as it shook the whole mankind and claimed large number of lives. The disease appeared as a threat against the human race. In order to save the existence of mankind, there was an urgent need to develop a weapon to fight this enemy of humanity. Multiple treatment options got started to be explored. Also, S.Muslim et al. suggested newer treatment option using rhACE2, angiotensin (1–7), and angiotensin (1–9) [17]. Since prevention is better than cure, vaccines definitely are a more preferred option over drugs used for treatment.

Seeing the urgency of the situation, there was a need to develop the vaccine as early as possible, and so, the vaccine was developed in a very small time frame. COVID-19 was first identified in December 2019 and by next December, i.e. December 2020, vaccines were available for mass vaccination [1, 18].

The UK Medicine and Healthcare product Regulatory Agency (MHRA) gave approval to ChAdOx1 nCoV-19 vaccine for large-scale vaccination of adults over 18 years in December 2020 [19].

Other vaccines which got approval in December 2020 were mRNA 1273 vaccine by Moderna and mRNA BNT 162b2 or mRNA BioNtech/ Pfizer Vaccine. These vaccines were approved by US FDA [20, 21].

Multiple other vaccines also followed. Janssen vaccine by Johnson and Johnson got US FDA approval in February 2021 [22].

ChAdOx1 nCoV-19 was developed by the Oxford University. It includes AZ1222, Covishield, Vaxzevria, and AstraZeneca vaccine. It is a recombinant adenovirus vector-based vaccine which encodes S-Protein of SARS-CoV-2. Four randomised controlled trials were conducted in different countries like South Africa, Brazil, and UK to establish the safety and efficacy of this vaccine. The vaccine was found to be safe and effective, and no increased incidence of thrombosis or thrombocytopenia was noted in these trials [23].

However, when mass vaccination programme was started, reports of increased incidence of thrombosis/thrombocytopenia were noted. The European Medical Agency (EMA) analysed the reports of 62 cases of CVST and 24 case of splanchnic vein thrombosis (SVT). Out of these, 18 cases had fatal outcome. EMA concluded that there is no direct link between thrombocytopenia and vaccination, and the only possible hypothesis is a condition similar to HIT (heparin-induced thrombocytopenia), and benefit of vaccination is more than the risk [24].

Greinacher et al. demonstrated that following vaccination with ChAdOx1 nCoV-19, there may be a development of a rare condition mediated by platelet-activating antibody directed against platelet factor 4 (PF-4) leading to immune thrombotic thrombocytopenia, and this condition clinically appears similar to autoimmune heparin-induced thrombocytopenia [25].

Schultz et al. reported cases of venous thrombosis and thrombocytopenia developing after ChAdOx1 nCoV-19 vaccine and proposed the term vaccine-induced thrombotic thrombocytopenia (VITT) for this condition, which is similar to heparin-induced thrombocytopenia [26].

One study reported the post mortem investigations of 18 persons who had died and had a recent history of COVID-19 vaccination. In this study, one case of myocarditis resulting into death was considered possibly associated with vaccination but could not be confidently proven. Two cases of death were likely because of VITT (vaccine-induced thrombotic thrombocytopenia), and in the remaining cases, no correlation with vaccination was found [27].

Myocarditis following vaccination has previously also been described after small pox vaccine and is supposed to be caused by autoimmune response [28].

There are multiple reports of myocarditis after COVID-19 vaccination.

One study reported an estimated incidence of 2.13 case/100,000 persons for myocarditis after BNT162b2mRNA vaccine. In this study, post vaccination myocarditis was more common in males, and most cases were mild to moderate in severity [29].

In the present study, most cases of death (18 out of 22 = 81.81%) were associated with CVST, thrombocytopenia/thrombosis/VITT, DIC, and haemorrhage. Three out of 22 (13.63%) cases had fatal cardiac adverse effects (myocarditis/myocardial infarction), and one (4.54%) patient died because of autoimmune hepatitis.

Majority of cases (18 out of 22 = 81.81%) had received ChAdOx1 nCoV-19 vaccines, 2 cases (9.09%) had received BNT162b2mRNA vaccine, and another 2 cases (9.09%) had received Janssen vaccine.

Nineteen out of 22 (86.36%) cases were between the age group 20 to 60 years. Thirteen out of 22 (59.09%) were females, and 9 (40.90%) were males.

Nine out of 22 (40.90%) had no significant medical history, while the remaining 13 cases (59.09%) had some previous medical history.

Most of the cases, 12 out of 22 (54.54%) developed symptoms or were admitted to hospital within 7 days of vaccination. Nine cases (40.90%) were noted in 2nd week and one case (4.54%) in 3rd week. Earliest onset of symptom was within 30 min (noted in one case).

Fifteen cases (68.18%) developed symptoms after 1st dose. In 7 cases (31.81%), the exact description of dose could not be made out.

Majority of death (14 out of 22 = 63.63%) took place within 1 week after vaccination/presentation/admission/intervention. Out of these 14 cases, 11 deaths occurred within 3 days. Two deaths (9.09%) occurred between 1 to 2 weeks and 3 deaths (13.63%) between 2 to 3 weeks. In 3 cases (13.63%), the exact time of death after presentation/vaccination/admission/intervention could not be identified, but these deaths took place during treatment for the complication developed after vaccination.

Analysis of these data indicates that persons who died had recent history of COVID-19 vaccination and probably succumbed to complications which developed in the temporal association of vaccination. Interestingly, many of these persons were previously healthy individuals and had no significant past medical history, though merely history of recent vaccination does not mean that these deaths occurred because of vaccination, but at the same time, it must be kept in mind that mankind till date has not found any magic bullet which will have only favourable effects and no adverse effects. So, a close watch must be kept on complications developing after vaccination (whether causal or association), and proper collection and analysis of post vaccination data regarding adverse effects must be done so that safety of vaccination can further be improved.

## Conclusion

Deaths noted in the present study cannot be definitely attributed to vaccination. However, a temporal association of death with vaccination does create a concern, which ultimately leads to hesitancy in vaccination. This panic and hesitancy can be overcome with proper understanding of fatal adverse conditions.

Information obtained from the present analysis will help in understanding the features of probable fatal adverse conditions in the temporal association of vaccination and will help the healthcare agencies to be prepared beforehand to tackle any untoward lethal event.

Understanding of fatal adverse conditions and preparedness to tackle these conditions will ultimately boost the safety of vaccination drive further.

### Disclaimer

The aim of this study is not to deter the vaccination programme, but it is to make the vaccination programme more safe by being aware of fatal adverse conditions in the temporal association of vaccination so that health care agency may remain prepared to tackle any untoward events.

## Data Availability

Not applicable.

## Abbreviations

CVST: Cerebral venous sinus thrombosis
VITT: Vaccine-induced thrombotic thrombocytopenia
DIC: Disseminated intravascular coagulation
HTN: Hypertension
NAD: Nothing abnormal detected
DM: Diabetes mellitus
NA: Not available
SAH: Subarachnoid haemorrhage
MI: Myocardial infarction
HT: Hashimoto thyroiditis
HRT: Hormone replacement therapy
VCR: Vaginal contraceptive ring
OCP: Oral contraceptive pill
Sx: Surgery
RT: Radiation therapy
ADT: Androgen deprivation therapy
AF: Atrial fibrillation
